# Effect of intensive education on stroke prevention and management ability of community doctors: a cross-sectional study

**DOI:** 10.1186/s12909-022-03125-z

**Published:** 2022-01-24

**Authors:** Xue-min Zhong, Yao Huang, Lanying He, Jian Wang

**Affiliations:** 1 Department of Neurology, The Second People’s Hospital of Chengdu, No. 10 South Qingyun Street, Chengdu, 610017 Sichuan China; 2 Department of Neurosurgery, The General Hospital of the Western Theater Command, 270 Rongdu Avenue, Jinniu District, Chengdu, 610083 Sichuan Province China

**Keywords:** Stroke, stroke prevention and treatment, Stroke management, Community doctors, Intensive education

## Abstract

**Background:**

Prevention and treatment of stroke are extremely important to reduce the incidence of stroke-related disability and the associated death. This study aimed to investigate the current ability of community doctors in stroke management in the Jinjiang district of Chengdu, China, and the effect of intensive education on stroke prevention and management ability of these doctors.

**Methods:**

A self-designed questionnaire was used to investigate the current status of stroke management by community doctors in the Jinjiang district. Subsequently, a series of intensive stroke management education courses for community doctors was designed according to the relevant guidelines for cerebrovascular accident prevention and treatment in China. All community doctors were trained, and their ability to treat and prevent stroke was reassessed using the self-designed questionnaire.

**Results:**

Of the 450 questionnaires issued, 370 (82.2%) and 389 (86.4%) community doctors were enrolled before and after intensive education, respectively. The results showed that only 37.8% of the community doctors in the Jinjiang district knew the guidelines for the prevention and treatment of cerebrovascular diseases, and only 45.9% thought they had stroke management ability. The stroke management ability of community doctors improved after intensive education (*p* < 0.05), including pre-hospital identification and management of stroke, and management of its risk factors.

**Conclusions:**

The capacity of community doctors in the Jinjiang district of Chengdu is far from meeting the requirements of stroke prevention and treatment. However, the stroke management ability of the community doctors was greatly improved by promoting intensive education.

**Supplementary Information:**

The online version contains supplementary material available at 10.1186/s12909-022-03125-z.

## Background

Ischaemic stroke was the cause of approximately 0.73 million deaths in China in 2016, accounting for nearly 40% of deaths from stroke, 70% of new-onset strokes, and 78% of the prevalence of stroke in China [1, 2]. Under the current community medical service model in China, community medical staff plays an important role in the stroke prevention and treatment system [3]. Presently, except for the relatively high quality of community medical staff in Beijing, Shanghai, and other developed areas, the theory and management ability of stroke of community medical staff in other areas are inadequate [4, 5]. A few studies have reported that the prevention and treatment ability of Chinese community doctors require improvement [3–5]. Only when these doctors fully grasp the principles of diagnosis and treatment can patients receive the most timely and appropriate treatment. The purpose of this study was to investigate the current standard of stroke management among community doctors in the Jinjiang district of Chengdu, to re-evaluate their ability after intensive education, and to determine the effectiveness of intensive education.

## Methods

### Sample

We conducted a survey of community doctors from nine communities (Chunxi, Yanshikou, Niushikou, Hongsha, Jinjiang, Lianxin, Wanke, Quan subtree, and Daci temple) in the Jinjiang district of Chengdu (China) from February 2017 to February 2019. According to the method for estimating the minimum sample size of quantitative data recommended by the Chinese Residents of Nutrition and Health Survey in 2002, 450 community doctors in Jinjiang district were considered an appropriate sample number and these doctors were randomly selected. A cluster sampling method was adopted, selecting 50 doctors in each community.

Ethical approval for this study was obtained from the Medical and Health Research Ethics Committee of the Second People’s Hospital of Chengdu, China. Written informed consent was obtained from all the participants. All methods were carried out in accordance with the pertinent literature on prevention and treatment of acute ischemic stroke (AIS) and the Chinese guidelines for the secondary prevention of ischemic stroke and transient ischemic attack (TIA) [6–11].

### Survey contents

Based on the Chinese guidelines for the prevention and treatment of AIS and the Chinese guidelines for the secondary prevention of ischemic stroke and transient ischemic attack (TIA) [6, 7, 9–11], a questionnaire on the current status of stroke management ability of community doctors was designed to conduct a cluster sampling survey among doctors from nine communities in the Jinjiang district. The questionnaire contained 23 questions, which were divided into three main sections:

1. Basic information: sex, age, education, title, specialty before engaging in community health services, general practitioner education status, time spent performing clinical work, engagement in community health services, among others.
2. Concepts related to early recognition and emergency treatment for stroke: pre-hospital stroke identification, assessment (including knowledge of stroke warning signs), and processing (awareness of thrombolytic therapy and its time window; airway management; assessment of circulation; monitoring of heart function, inhaled oxygen supply, and blood glucose; establishment of intravenous route; and transfer of patients to the nearest comprehensive stroke center as soon as possible).
3. Knowledge about secondary stroke prevention: risk factors for stroke; definition and management of TIA; general and ideal goal for target blood pressure, HbA1c, and international normalized ratio levels; and awareness of statin, warfarin, and antiplatelet therapies and their side effects. We conducted stroke health education activities with the theme “understanding stroke” and evaluated the outcome. We designed a series of intensive stroke management education courses for community doctors according to the relevant guidelines for cerebrovascular prevention and treatment in China [6–8], including the management of major modifiable risk factors for stroke (hypertension, smoking, diabetes, carotid stenosis, atrial fibrillation, dyslipidemia, lack of physical exercise, unhealthy dietary habits, alcohol consumption, drug abuse, and obesity).

Intensive education was conducted through face-to-face lectures, online education, and the distribution of handouts and information manuals by experts in the area. The doctors were reassessed before and after the intensive education, using the self-designed questionnaire to determine whether the education could improve their stroke management ability.

### Statistical analysis

After sorting out the data processing and survey data, the data were entered into the EPIDATA database and then imported into SPSS version 22 (IBM Corp., Armonk, NY, USA) for statistical analysis. Descriptive statistical analysis was used to assess the general characteristics of respondents, identify stroke-related risk factors, identify warning symptoms of stroke, and identify management ability of stroke. Chi-square test was used to analyze the general characteristics of doctors before and after intensive education and the doctors’ knowledge of stroke-related knowledge and management ability of stroke before and after intensive education. Continuous and normally distributed variables were expressed as the mean ± standard deviation, and variables not normally distributed were expressed as medians (interquartile ranges). Categorical data were described as frequencies and percentages. *P* value less than 0.05 was regarded as statistically significant.

## Results

### Questionnaire responses

In total, 450 questionnaires were issued before intensive education, 370 of which were returned, indicating a valid response rate of 82.2%. After the intensive education, 450 questionnaires were reissued, 389 of which were returned, indicating a valid response rate of 86.4%.

### General information of respondents

In total, 370(82.2%) and 389(86.4%) community doctors were enrolled before and after intensive education, respectively. The general information (sex, age, educational level, professional title, specialty before engaging in community health services, general practitioner education status, education time, clinical experience time, and community service experience time) of the respondents before and after intensive education showed no significant difference between the two groups (Table 1).

**Table 1:** General information of respondents

	Before intensive education (*n* = 370)	After intensive education (*n* = 389)	*p* values
Sex (%)			0.622
Male	109 (29.5)	121 (31.1)	
Female	261 (70.5)	268 (68.9)	
Age (X ± S, y)	33.0 ± 9.6	34.2 ± 10.1	0.894
Education level			
Technical secondary school graduates and below	96 (25.9)	94 (24.2)	
Bachelor	216 (58.4)	228 (58.6)	
Masters or higher degree	58 (15.7)	67 (17.2)	
Job title			0.918
Resident	165 (44.6)	172 (44.2)	
Attending physician	162 (43.8)	168 (43.2)	
Deputy or chief physician	43 (11.6)	49 (12.6)	
Specialty before engaging in community health services			0.436
Internal medicine	228 (61.6)	241 (62.0)	
Surgery	31 (8.4)	42 (10.8)	
Others	111 (30.0)	106 (27.2)	
GP education	181 (48.9)	204 (52.4)	0.332
education time (mo)	3–6	3–6	1.000
Clinical experience time (y)	2.5–15.5	3–15	1.000
Community experience time (y)	2–10	2–10	1.000

*GP* general practitioner

### Stroke management ability of community doctors before intensive education

The results of our study showed that only 7.0% (26/370) of the community doctors had knowledge of all the five warning symptoms of a sudden stroke, and 34.9% (129/370) did not know any of the symptoms. Furthermore, only 3% (11/370) of the respondents correctly described the pre-hospital management of acute stroke. Although most doctors knew that thrombolysis is the most effective treatment for AIS, less than half [40.3% (149/370)] knew the thrombolytic time window. Only 20.8% (77/370) named four or more risk factors for stroke, and approximately 30% (108/370) were unable to name any. Less than 50% knew how to properly manage the risk factors for stroke, such as hypertension, diabetes, and atrial fibrillation.

### Stroke management ability of community doctors after intensive education

After intensive education, 13.1% (51/389) of the community doctors correctly mentioned all five warning symptoms of stroke, while 20.3% (79/389) could not mention any; and 28.0% (109/389) correctly described the pre-hospital management of acute stroke. The proportion of doctors who knew that the most effective treatment for AIS was thrombolytic therapy, along with the knowledge of its time window, increased to 61.0% (214/389). Regarding stroke prevention, 25.2% (98/389) of the community doctors named five risk factors for stroke, which was better than before (*P*<0.001), while 13.9% (54/389) could not name any. The proportion of community doctors who knew how to properly manage the risk factors for stroke, such as hypertension, diabetes, and atrial fibrillation, was also significantly higher (*P*<0.001). The results showed that the stroke management ability of community doctors improved after intensive education (Table 2).

**Table 2:** Effect of intensive education on the stroke management ability of community doctors

N (%)	Before intensive education (N = 370)	After intensive education (N = 389)	***p***
Prevention and treatment of cerebrovascular diseases			
Knowledge of relevant guidelines	140 (37.8)	265 (68.1)	< 0.001
Classification of stroke	301 (81.4)	351 (90.2)	< 0.001
Knowledge of stroke warning signs			< 0.001
0	129 (34.9)	79 (20.3)	
1	62 (16.8)	91 (23.4)	
2	49 (13.2)	42 (10.8)	
3	63 (17.0)	51 (13.1)	
4	41 (11.1)	75 (19.3)	0.918
5	26 (7.0)	51 (13.1)	
Pre-hospital stroke assessment methods	9 (2.4)	109 (28.0)	
Management of acute ischemic stroke in community health centers (stations)			< 0.001
0 score	289 (78.1)	201 (51.7)	< 0.001
1 score	26 (7.0)	50 (12.9)	
2 score	21 (5.7)	41 (10.5)	
3 score	13 (3.5)	37 (9.5)	
4 score	10 (2.7)	28 (7.2)	
Score ≥ 5	11 (3.0)	32 (8.2)	
Thrombolytic therapy for acute ischemic stroke	329 (88.9)	351 (90.2)	0.554
Time window for thrombolytic therapy	149 (45.3)	214 (61.0)	< 0.001
Risk factors for stroke			< 0.001
0	108 (29.2)	54 (13.9)	
1	30 (8.1)	25 (6.4)	
2	44 (11.9)	28 (7.2)	
3	72 (19.5)	97 (24.9)	
4	77 (20.8)	87 (22.4)	
Score ≥ 5	39 (10.5)	98 (25.2)	
Definition of TIA	159 (43.0)	202 (51.9)	0.014
TIA management in community health centers	51 (13.8)	105 (27.0)	< 0.001
General goal for target BP level for the prevention of recurrent stroke	247 (66.8)	289 (74.3)	0.023
Ideal goal for target BP level for the prevention of recurrent stroke	170 (45.9)	215 (55.3)	0.010
Goal for target HbA1c level for the prevention of recurrent stroke	152 (41.1)	239 (61.4)	< 0.001
Goal for BP for the prevention of recurrent stroke in hypertensive patients with diabetes	152 (41.1)	239 (61.4)	< 0.001
Awareness of statins therapy for the prevention of recurrent stroke	199 (53.8)	301 (77.4)	< 0.001
General goals for LDL-c level for the prevention of recurrent stroke	28 (7.6)	107 (27.5)	< 0.001
Side effects of statins			0.296
abnormal liver function	45 (12.2)	103 (26.5)	
muscle damage	30 (8.10	89 (22.9)	
knowing both side effects	41 (11.1)	78 (20.1)	
Goals for LDL-c level for the prevention of recurrent stroke in patients associated with multiple risk factors or evidence for atherosclerotic vulnerable plaque or arterial embolism	31 (8.4)	95 (24.2)	< 0.001
Awareness of warfarin therapy for the prevention of recurrent stroke in patients with atrial fibrillation	165 (44.6)	278 (71.5)	< 0.001
Goal for INR level in patients who receive warfarin therapy	108 (65.5)	217 (78.1)	0.004
Awareness of antiplatelet therapy for the prevention of recurrent stroke	88 (23.8)	189 (48.6)	< 0.001

*TIA* transient ischemic attack, *BP* blood pressure, *LDL-C* low-density lipoprotein cholesterol, *INR* international normalized ratio

## Discussion

Previous surveys have reported that the stroke management ability of community doctors in China was low and required immediate improvement [3–5, 12, 13]. A study of Botswana’s community physicians also suggested that community physicians may be less likely to contribute to stroke [14]. Our survey found that only 37.8% of community doctors in the Jinjiang District of Chengdu knew the guidelines for the prevention and treatment of cerebrovascular diseases, and only 45.9% thought they had stroke management ability. The results of our study indicated that community doctors in the Jinjiang district of Chengdu lacked the ability to manage the identification and pre-hospital treatment of stroke and the secondary prevention of stroke recurrence.

This is the first study to present results on intensive education of community doctors on stroke prevention and their management ability in stroke treatment. The community doctors are the health advisors of the vast number of community residents. Only when the community doctor is familiar with the recognition, treatment, and referral of stroke, can more patients with stroke benefit.

Stroke is one of the diseases that cause human disability and death, and AIS accounts for 80% of all strokes [1]. Numerous studies have shown that improving modifiable risk factors for stroke, including hypertension, diabetes, hyperlipidemia, and smoking, can reduce the incidence of the disease [15–18]. Compared with before intensive education, the proportion of community doctors who knew how to properly manage the risk factors for stroke, such as hypertension, diabetes, and atrial fibrillation, was also significantly higher (*P*<0.001).

The National Stroke Registry of China reported that 17.7% of 11,560 patients with ischemic stroke or TIA experienced a recurrence within 1 year [19]. Rapid identification and treatment of TIA are important to prevent recurrence of cerebral infarction. Through intensive education, the proportion of doctors who could correctly manage TIA increased significantly (*P*<0.001).

The main method of AIS management is intravenous thrombolysis, and the earlier the time, the greater the benefit of patients [8]. After intensive education, the proportion of doctors who knew the most effective treatment was thrombolytic therapy and knew the time window of thrombolytic therapy increased significantly (*P*<0.001). Our results showed that the stroke management ability of community doctors increased after intensive education. After the education, 68.1% of community doctors in the Jinjiang District of Chengdu knew the guidelines for the prevention and treatment of cerebrovascular diseases, and 81.5% thought they had stroke management ability. Hence, this intensive education is helpful for the prevention, recognition, treatment, referral, and rehabilitation of patients with stroke.

The shortcomings of this study are as follows: (1) the scope of the survey is limited to the Jinjiang District of Chengdu, the sample size was small, and the results do not reflect the overall situation of Chengdu; (2) compared with open-ended questions, the closed questions used in this survey may have resulted in a higher response rate in the evaluation of the ability to master stroke-related knowledge; and (3) not all knowledge of stroke management was assessed in the test questionnaires.

## Conclusions

In summary, the capacity of community doctors in the Jinjiang district of Chengdu is far from meeting the requirements of stroke prevention and treatment before intensive education. Intensive stroke education of community doctors proved to be beneficial in improving their theoretical knowledge and clinical management of stroke. This can help in improving the diagnosis and treatment capacity of community health centers, thereby achieving the goals of reducing the incidence and mortality of stroke. Therefore, intensive education is worth promoting.

## Supplementary Information

**Additional file 1.**

## Data Availability

The datasets used and/or analyzed during the current study are available from the corresponding author on reasonable request.

## Abbreviations

AIS: Acute ischemic stroke
MR-CLEAN: Multicenter randomized clinical trial of endovascular treatment for acute ischemic stroke in the Netherlands
TIA: Transient ischemic attack
