# Supplementary material for: Effect of intensive education on stroke prevention and management ability of community doctors: a cross-sectional study
Source: BMC Med Educ. 2022 Jan 24;22:53. doi: 10.1186/s12909-022-03125-z (PMC8788122; doi:10.1186/s12909-022-03125-z)
Supplement: Supplementary file 1 — Additional file 1. [file 12909_2022_3125_MOESM1_ESM.docx]

**Survey of community medical staff's knowledge about stroke prevention**

Basic Information

1. Gender: □male □female

2. age:

3. Education: □ Technical secondary school and below □ Associate □ Undergraduate □ Master and above

4. Title: □Resident physician or equivalent professional title □ Attending physician or equivalent professional title □Deputy Chief Physician or equivalent title □ Chief Physician or equivalent title

5. Majors before engaging in community health services: □Internal medicine □Surgery □Gynecology and obstetrics □Pediatrics □Psychiatry □Optometrists □Emergency department □Laboratory and ultrasound □Dermatology □General medicine □Chinese medicine □Public health □Others

6. Have you received general practitioner training: □Yes □No Training time: _______ months

7. Time engaged in clinical work: _______ years

8. Engaged in community health services: _______ years

9. Do you think you have or undertake the ability to manage stroke patients: □Yes □No

Part Two Concepts related to stroke, early recognition and emergency treatment

10. Do you know the relevant guidelines for the prevention and treatment of cerebrovascular diseases in China: □Yes □No

11. What are the two types of stroke: ________________________.

12. Please list, when the patient suddenly appears what symptoms suggest the occurrence of stroke?

13. Do you know the prehospital assessment methods for stroke? Please describe the method.

14. When a community health service center (station) receives patients with acute stroke, how should they perform emergency treatment?

15. In the treatment of acute ischemic stroke, the most effective treatment method is ____________, and its effective time window is ____________.

Part Three Knowledge about Stroke Prevention

16. What are the main (controllable) risk factors for stroke ___________________________ ____________________________________________________________________.

17. Individuals who are suitable for the use of antiplatelet aggregation drugs (such as aspirin) for primary stroke prevention are _______________________________________________________________________.

18. It is recommended that patients with asymptomatic carotid artery stenosis take ____________ and ____________ drugs daily to screen for other treatable stroke risk factors, carry out reasonable treatment and change their lifestyle.

19. For patients with prehypertension (systolic blood pressure_______diastolic blood pressure_______), it is recommended to screen for hypertension every year and promote a healthy lifestyle; hypertensive patients need antihypertensive therapy, and the target blood pressure should be lower than _______.

20. Patients with dyslipidemia determine the target value of blood lipids based on ____________. For patients with a 10-year risk of cardiovascular events, ____________ should be performed first, and blood lipids should be reviewed regularly. In addition, ____________ is recommended for primary prevention of ischemic stroke.

Patients with dyslipidemia, hypertension, diabetes, and cardiovascular disease are at high risk/very high risk of stroke. Regardless of their baseline LDL-C level, these patients are encouraged to adopt lifestyle changes and statin therapy to reduce LDL-C to_ ___________ or lower LDL-C level from baseline ____________.

The main side effects of statins are _______ and _______, which should be monitored regularly.

21. The blood pressure of diabetic patients should be strictly controlled below ________, which can be stratified according to its risk

And the tolerance is further reduced. On the basis of strict control of blood sugar and blood pressure, diabetics can effectively reduce the risk of stroke in combination with ________.

twenty two. Patients with valvular atrial fibrillation with CHA2DS2-VASc score _________ have a higher risk of stroke and a lower risk of bleeding complications. It is recommended to take long-term oral warfarin anticoagulant therapy, and the target INR is _________. For non-valvular atrial fibrillation patients with CHA2DS2-VASc score _________, the risk of bleeding complications is low, and oral anticoagulant therapy is recommended. Choices include _________ or _________.

23. For patients with non-cardiac embolic ischemic stroke or TIA, it is recommended to use _______ to reduce the recurrence of ischemic stroke and TIA. _______ (dose_______) and _______ (dose_______) can be the first choice
